# The Longer You Stay, the Worse Your Health? A Critical Review of the Negative Acculturation Theory among Asian Immigrants

**DOI:** 10.3390/ijerph110808038

**Published:** 2014-08-08

**Authors:** Annie Ro

**Affiliations:** Program in Public Health, University of California, Irvine, 653 E. Peltason Dr., CA 92697, USA; E-Mail: annie.ro@uci.edu; Tel.: +1-949-824-6185; Fax: + 1-949-824-0529

**Keywords:** Asian immigrants, acculturation, physical health, critical review

## Abstract

Researchers have become increasingly interested in the health patterns of immigrants with longer residence in the United States, as this reveals the health consequences of integration processes. The negative acculturation effect has been the dominant interpretation of duration patterns, despite empirical and theoretical uncertainties about this assumption. This theory assumes that immigrant health declines with longer residence in the United States because of poorer health behaviors and health risks that reflect Americanized lifestyles. This paper reviews the empirical support for the negative acculturation theory among Asian immigrants to determine if and when it is an appropriate interpretation for duration patterns. I conclude that empirical inconsistencies and methodological issues limit the negative acculturation theory as the primary interpretation for duration patterns. First, there is no consistent evidence that health behaviors decline with time. There is also substantial group heterogeneity in duration patterns as well as heterogeneity across health outcomes. The literature has not adequately addressed methodological shortcomings, such as confounding by cohort effects or non-linear duration patterns. Length of residence in the United States is still an important aspect of Asian immigrant health, but the mechanisms of this relationship are still understudied. I propose alternative frameworks between duration and health that consider environmental influences and end with future research directions to explore research gaps.

## 1. Introduction

It is widely acknowledged that immigrant health deteriorates with longer residence in the United States. This pattern, often called “the negative acculturation effect”, is assumed to be driven by unhealthy cultural changes towards Western lifestyles [1]. Several researchers have drawn attention to the uneven empirical support for this trend, however [2]. The associations between longer duration and poorer health are found in some health outcomes [3,4] but not others [5], and in some immigrant groups but not others [4,6]. The goal of this review paper is to conduct a thorough accounting of the empirical support for the negative acculturation theory among Asian immigrants. Despite empirical variation, negative acculturation theory dominates the literature as the primary mechanism underlying duration and health patterns. By highlighting patterns and inconsistencies, I aim to clarify if and when the negative acculturation theory is an appropriate interpretation for duration patterns. I also propose more complex integration patterns beyond the lifestyle and behavioral changes implied in the negative acculturation theory. 

The negative acculturation theory interprets duration patterns through the lens of cultural integration; those who have longer residence in the United States have more exposure to and are thus more integrated into its social environment. This process largely follows Gordon’s classic Anglo-Conformity framework, which posits change on the part of the immigrant group towards middle-class Anglo culture [7]. As a result, the health impacts of acculturative processes have been interpreted through individual-level behavioral changes that represent the extent to which immigrants adopt “Western” lifestyles. 

Specifically, immigrants adopt unhealthy diets and norms toward health risks and shed health-protective ethnic resources, such as cultural practices and ethnic diets [8]. In the case of obesity, for example, immigrants’ dietary preferences change from ethnic foods to diets that are higher in fat, more processed, and contain more meat [9]. The high rates of obesity in the United States represent a population-level trend that immigrants gradually reflect as they acculturate [4]. There is also an environmental component to this process; the obesogenic American environment produces certain obesity risks that accumulate with time among immigrants [10]. These forces serve to worsen immigrants’ health and we thus assume that Americanization and its associated processes have a net negative impact on immigrants’ health status. When applied to duration research, this means that immigrants with longer residence should display poorer health than those who have immigrated more recently.

This view has not been without its detractors. First, the relevance of acculturation itself in immigrant health has been contested. In their critique of acculturation in Latino health research, Hunt *et al.* [11] argue that cultural boundaries are too fluid to warrant substantial change from one culture to another. They also question the unidimensional and linear nature of Gordonian assimilation processes, arguing that immigrants have complex ways of adapting to their new environments. Likewise, Abraido-Lanza, Armbrister, Florez and Aguirre [8] argue that studies of acculturation and health tend to espouse a simplistic view of cultural change that does not reflect more sophisticated views of acculturation that have been developed in the broader social sciences. Viruell-Fuentes [12] caution that the predominance of individually-driven cultural change as a “catch-all” construct decontextualizes immigrant health from environmental processes related to economics, history, and politics. 

The exact mechanisms and pathways to health that are encompassed within duration have also been subject to interpretation [2]. Years in the United States is not itself a causal health factor, but provides the situational context in which certain health risks appear. While the prevailing arguments in negative acculturation theory identify health behaviors as the driving force in duration patterns [13], other interpretations highlight social interactions or English ability [14]. Still others suggest that duration represents the accumulation of environmental stressors, such as acculturative stress, discrimination, or work stress [15,16]. Part of the uncertainty regarding mechanisms stems from the inexact measurement of acculturation itself. Several reviews have catalogued the array of scales and proxy measures that have been utilized to measure acculturation, which reflect its diverse conceptualization [2,17,18]. 

In light of these debates, a review can ascertain the extent to which empirical work supports the negative acculturation theory, both in its hypothesized mechanisms as well as in the expected negative relationship between health status and duration. This paper focuses on Asian immigrants for several reasons. Most of the data on duration and health has been conducted among Latino immigrants, yet the negative acculturation theory is often applied to immigrants at large. Asian immigrants may not only differ from Latinos in duration trends, but their unique patterns can underscore the role of cultural differences or circumstances of migration. The growing Asian immigrant population also has heterogeneous health patterns and socioeconomic profiles across ethnic groups. This review also considers differences in duration patterns across Asian ethnicities where applicable. While the literature on Asian populations may be growing, it still remains within a reasonable scope to provide an overview of the field. It may be prudent to start a thorough review with Asian immigrants to highlights potential gaps and areas for future research, which can then be applied to other larger immigrant groups such as Latinos. 

The paper begins with a review of the published literature on duration and health outcomes among Asian immigrants. The first focus is on health behavior outcomes, as they are the primary mechanisms of the negative acculturation theory. The paper then considers duration patterns in four physical health outcomes: body weight, chronic conditions, disability and self-rated health. After reviewing the literature, I discuss the larger implications of the findings and propose areas for future research.

## 2. Methods

The papers included in the review were located through a key word search using the following terms and Boolean operators: “Asian immigrants” AND (“duration” OR “length of residence”) AND “acculturation” AND “health” in the PubMed and Google Scholar journal databases. There was no limit on the article publication year. I only included those articles that used U.S. data and examined the relationship between years in the United States and a physical health or health behavior outcome in a population-based sample. Most of the articles conducted multivariate analyses, although a few had bivariate associations with age adjustment only. I only included studies with Asian samples, either aggregated into a single group or analyzed separately by Asian ethnicity. I excluded studies that grouped Asian immigrant respondents with other immigrant groups (*i.e.*, Latino) and controlled for race or region/country of origin or ran interactions by duration and race/ethnicity. I also excluded studies that measured length of stay in the United States through other measures, such as age at migration or proportion of life spent in the United States. While these are important constructs, their theoretical implications include developmental processes that are beyond the scope of this review. I also searched the citations of two frequently cited papers on duration and health among Asian immigrants and identified additional articles using the same criteria [3,19]. After eliminating articles that did not meet the review criteria, the final number of studies was 28 (Table 1). Unless otherwise noted, all of the studies included in the review utilize cross-sectional data. 

**Table 1:** Empirical papers included in review.

Reference	Data	Sample Size	Outcome and Duration Effect
**Health Behaviors**			
Frisbie, Cho, Hummer (2001) [3]	1992–1995 NHIS	8249	Annual visits to physician: Positive No access to health care: Positive
Kandula & Lauderdale (2005) [20]	2001 CHIS	29,473 (4226 Asian)	Leisure time physical activity: Positive Non-leisure time physical activity: Negative Physical inactivity: Positive (women)
Maxwell *et al.* (2005) [21]	2001 CHIS	3956	Current Smoking: No effect
Dey, Wilson, Lucas (2006) [22]	1998–2003 NHIS	5379	Current Smoking: Negative
Taylor *et al.* (2007) [23]	Community sample of Chinese residents in Seattle	1509	Fruit/vegetable consumption: Positive Physical activity: Positive Tobacco use among men: No effect Cholesterol Check: Positive Blood Pressure Check: No effect
An, Cochran, Mays (2008) [24]	2001–2003 CHIS	8192	Current Smoking: Positive (women) Quitting: No Effect
Akresh (2009) [25]	New Immigrant Survey 2003	2772	Visiting physical at all: Positive Hospital as primary source of care: No effect Visiting Dentist: Positive Homeopathic medicine: Positive
Osypuk, Diez Roux *et al.* (2009) [26]	Multi-ethnic study of Atherosclerosis (MESA) Chinese sample	711	High fat and processed meat diet: No effect
Chou, Johnson, Blewett (2010) [27]	1998–2004 NHIS Chinese sample	1217	ER Visits: Negative Smoking: No effect
**Body Weight**			
Lauderdale & Rathouz (2000) [28]	1992–1995 NHIS	7263	Overweight: Positive Obesity: Positive
Goel *et al.* (2004) [4]	2000 NHIS	846	BMI: Positive
Cho & Juon (2006) [29]	2003 CHIS Korean Sample	492	Overweight/Obesity: Positive
Kaushal (2009) [30]	1990–2004 NHIS	7672	Obesity: No relationship
Oza-Frank & Narayan (2009) [6]	NHIS 1997–2005	1013 South Asians 1651 Central Asians 2139 South East Asians	Overweight Positive (Central Asians) No relationship (South and Southeast Asians)
Yeh *et al.* (2009) [31]	Community sample of Chinese Americans in New York city	2342	BMI: Positive
Albrecht *et al.* (2013) [32]	Multi-ethnic study of Atherosclerosis (MESA) Longitudinal Chinese sample	1002	BMI: No relationship Waist Circumference: Positive
Ro *et al.* (2013) [33]	1995–2009 NHIS	44,002	Obesity: Positive
**Chronic Conditions**			
Singh & Miller (2004) [34]	1992–1995 NHIS	--	Chronic Disease Prevalence: Positive
Dey, Wilson, Lucas (2006) [22]	1998–2003 NHIS	5379	Diabetes: Inverse Hypertension: Positive
De Castro, Gee, Takeuchi (2008) [16]	Community sample of Filipino Americans	2285	Chronic health conditions: Positive
Gong, Xu, Takeuchi (2011) [35]	NLAAS	2095	Physical Discomfort: No effect
**Disability**			
Cho & Hummer (2001) [19]	1990 Census	201,828	Work: Positive Mobility: Negative (Older ages) Positive (Younger ages) Self-care: Inverse
Frisbie, Cho, Hummer (2001) [3]	1992–1995 NHIS	8249	Activity limitations: Positive Bed days (1–6 days): Positive Bed days (Over 1 week): Positive
Singh & Miller (2004) [35]	1992–1995 NHIS	--	Disability days: Positive Activity limitations: Positive
Dey, Wilson, Lucas (2006) [22]	1998–2003 NHIS	5379	Annual bed days: Positive
Ro and Gee (2010) [36]	2000 Census	294,967	Mental: Positive (Younger ages) Self-care: No relationship Sensory: Positive (Younger ages) Physical: Positive (Younger ages) Mobility: Negative (All ages) Positive (Younger ages) Work: Positive (Older ages) Any Disability Negative (All ages) Positive (Younger ages)
**Self-Rated Health**			
Frisbie, Cho, Hummer (2001) [3]	1992–1995 NHIS	8249	Fair/Poor Self-Rated Health: Positive
Dey, Wilson Lucas (2006) [22]	1998–2003 NHIS	--	Fair/Poor Self-Rated Health: Inverse
Zhang & Ta (2009) [37]	NLAAS	2095	Fair/Poor Self- Rated Health: Positive
Acevedo-Garcia, Bates, Osypuk & McArdle (2010) [38]	2003–2007 CPS	22,804	Fair/Poor Self-Rated Health: Positive
Chou, Johnson, Blewett (2010) [26]	1998–2004 NHIS Chinese sample	1217	Fair/Poor Self-Rated Health: Inverse
Ihara (2011) [39]	2011 CHIS	4,716	Fair/Poor Self-Rated Health: No Effect
Gong, Xu, Takeuchi (2011) [35]	NLAAS	2095	Fair/Poor Self-Rated Health: Positive
John, de Castro, Martin, Duran, Takeuchi (2012) [40]	NLAAS	1530	Fair/Poor Self-Rated Health: No Effect

## 3. Results

### 3.1. Health Care Utilization/Access and Health Behaviors

Health behaviors are a key mechanism of the negative acculturation theory. One area of the health behavior literature examines health services use with longer duration in the United States. In general, it appears that immigrants visit the doctor more and have more access to care with longer duration. Using the National Health Interview Survey (NHIS), Frisbie *et al*. [3] found that shorter term immigrants (0–5 years and 6–10 years duration) were significantly less likely see a doctor annually or to visit a doctor more than three times a year compared to U.S. born Asians. Those with over 10 years residence did not differ from the US-born in this regard. Likewise, those with under 10 years of residence were more likely to say they had no access to health care, while those over 10 years did not significantly differ from the U.S.-born. In the New Immigrant Survey, Akresh [25] similarly found that years in the United States was positively linked to visiting a doctor at all in the past year. Years in the United States was analyzed as a quadratic function and the positive effect weakened after 14–17 years. Among Chinese immigrants, emergency room (ER) visits declined with duration, perhaps highlighting better access to preventative medical care [27].

The implications of more medical access are not clear. More doctor visits might imply improving health with duration as immigrants receive better medical care and management. Alternatively, immigrants could be visiting doctors more often because they are sicker. Further, we do not know the purposes of the visits or which procedures were conducted. For example, one study found Chinese immigrants with over 10 years of residence in a representative sample in Seattle had a higher likelihood of cholesterol checks, but not blood pressure checks [23]. Finally, these studies do not give us any indication of the quality of care or the extent of medical attention. Immigrants overall are less satisfied with the medical encounter than the US-born [41]. More frequent doctor visits may not translate into better health if immigrants are dissatisfied by the clinical interaction or do not have their medical needs addressed appropriately.

In regard to diet, the empirical evidence is mixed and does not indicate unhealthier lifestyles. The consumption of both healthy and unhealthy foods increased with longer duration. Among a representative sample of Chinese immigrants in Seattle, longer term immigrants ate more fruits and vegetables than recently-arrived immigrants [23]. Another study of Chinese immigrants in Los Angeles and Chicago found an increase in diets higher in fat and processed meats across duration, however [26]. 

The relationship between duration and physical activity was mixed as well. Longer-tem Chinese immigrants in Seattle reported more physical activity than their more recent counterparts [23]. Kandula and Lauderdale [20] further distinguished among types physical activity among a representative sample of Californian immigrants (leisure time, non-leisure time and physical inactivity) and also found a significant increase in leisure time physical activity. For women, however, physical inactivity also increased with duration. 

Finally, there is no strong indication that smoking is related to duration. Of the four smoking studies, only one found an increase in smoking with longer duration among Chinese immigrants, but for women only [27]. 

### 3.2. Body Weight

We now consider the direct relationship between duration and physical health outcomes. Body weight had the most consistent support for the negative acculturation effect; of the eight studies that examined the change in BMI or overweight/obesity status by duration, six found support for increasing BMI or higher odds of overweight/obesity with increasing years in the United States. While each study included a unique set of covariates, it seems that the duration patterns were robust to controls for demographic characteristics such as age, gender, education, marital status, employment and Asian ethnicity. What is more, the duration patterns were not explained by potential mediating factors, such as health behaviors (alcohol, smoking, physical activity) and occupational status [4,6,32,33]. 

The duration and body weight relationship differed across certain groups. Lauderdale and Rathouz [28] found that men had higher odds for being overweight with longer duration while women had higher odds for obesity. There were also differences across Asian ethnic groups. Specific Korean and Chinese immigrant communities displayed a positive relationship between duration and body weight [29,31]. Oza-Frank [6] also found a duration effect for obesity among immigrants from Central Asia (*i.e.*, China) but not from those from Southeast Asia or South Asia. 

The two null studies had unique analysis features. The first null study did not find any significant duration effects in obesity after controlling for cohort of entry [30]. Because duration and cohort are confounded in cross-sectional data, these findings suggest that some of the differences across duration groups in previous studies may be due to compositional differences. In contrast, another study [33] found significant increases in obesity controlling for cohort of entry, suggesting that duration is still an important driver of obesity trends even after accounting for compositional differences across cohorts. These initial investigations indicate that compositional differences across cohorts can account for some of the duration differences, but whether they fully explain duration patterns is still unknown. 

The second null study was the only one to utilize longitudinal data (and hence did not have the cohort/duration confounding issue) and had mixed findings [32]. In this study, Chinese immigrants did not display significant increases in BMI over a 5 year follow-up period. They did, however, display higher waist circumference over time, but these differences were not statistically larger than those for the US-born. Physical activity, smoking status, and alcohol use did not mediate any of the results. 

Nearly all studies found a gradient increase in mean BMI or odds of overweight/obesity over dummied duration categories. One study, however, found an inverted-U shape; predicted BMI peaked at the middle duration immigrants (10–14 years) then decreased for the longest term immigrants (15+ years) [29]. This pattern contrasts with the linear assumption of the negative acculturation theory, suggesting that there are duration periods in which immigrants’ health is particularly vulnerable. 

### 3.3. Chronic Conditions

Two studies examined self-reported chronic conditions and both found that longer term immigrants had higher odds for reporting prevalence of any chronic disease. Both studies examined specific Asian ethnic groups. The de Castro, Gee and Takeuchi [16] study used a representative sample of Filipinos living in San Francisco and Honolulu and measured years in the United States as a continuous variable. The Singh and Miller study [34] utilized the NHIS and divided the Asian American sample into ethnic groups. Within each subgroup, they compared immigrants with under and over 15 years duration and found higher odds for chronic disease prevalence among the longer-term Chinese, Japanese, Filipinos and other API immigrants (the Korean, Asian Indians and Vietnamese longer-term immigrants were combined with the U.S. born to increase sample size, and we are thus unable to draw conclusions about the duration effect for these groups). 

When examining specific chronic diseases, however, there is less consistency. For example, physical discomfort was not associated with duration [35]. Physical discomfort is not a chronic illness per se, but is a possible symptom of underlying chronic conditions. Dey and Wilson Lucas [22] found a positive duration effect for hypertension but an inverse relationship between diabetes and length of U.S. residence. Methodological weaknesses limit this study, however, as the analysis consisted of a means comparisons only adjusting for age. Despite the methodological shortcomings, the discrepancy between the general chronic disease reports and specific diseases raises the possibility that the health risks encompassed by duration produce unique risks for certain health outcomes and not others.

### 3.4. Disability

The duration patterns for disability varied upon on the measure. There was consistent evidence of increasing disability with longer duration when assessed by activity limitations and bed days [3,22]. Similar patterns were found for bed days and activity limitations for individual Asian ethnicities, including Chinese, Japanese and Filipino [34]. 

Other disability measures were moderated by age. In the 1990 Census, work disability did not appear to have a duration pattern, but when age moderation was considered, a significant duration effect was present at younger ages [19]. For mobility limitations, the youngest immigrants actually displayed an inverse pattern whereby the longest-term immigrants had the lowest odds of mobility limitations. This reversed at older ages, where longer-term immigrants showed higher disability. Self-care had an inverse relationship; those with the longest duration reported the lowest odds. This was constant across all age groups.

A similar analysis conducted in the 2000 Census also found age moderation, but the patterns differed from the 1990 data [36]. For example, a duration effect in work disability was only present at older ages. Mobility disability displayed an inverse pattern such that those with the longest duration had the lowest odds relative to U.S.-born Asians. When stratifying by age, however, the youngest immigrants displayed a positive pattern whereby the longest-term immigrants had the highest odds. Self-care displayed an inverse relationship. The 2000 Census included sensory, physical and mental disability. These measures all displayed a similar pattern in which the prevalence rose with duration for younger immigrants only. The significant, but inconsistent, age moderation leaves us with few conclusions about its effect on disability.

### 3.5. Self-Rated Health

The evidence for declining self-rated health with increasing years is decidedly mixed. Four out of the eight studies found worsening self-reported health with longer duration [3,35,37,38]. Of those that found a significant relationship, differences among the duration categories were weak and did not support a linear trend. All studies modeled duration as a series of dummy variables (*i.e.*, 0–5 years, 6–10 years, 11–14 years, 15+ years). The duration categories followed a threshold pattern, such that duration groups under a certain number of years had better self-rated health than groups with more years, but did not differ from one another. This pattern indicates a shift in self-rated health that occurs soon after migration, rather than a gradual or cumulative trend as is implied by the negative acculturation theory. 

Three studies actually found an inverse effect, such that longer-term immigrants reported improving self-rated health compared to their more recent counterparts [22,27,33]. The Ro *et al.* paper [33] utilized the NHIS and found improving self-rated health with duration after controlling for cohort effects. When self-rated health patterns were examined within individual cohorts, the oldest cohort (*i.e*., those entering before 1980) displayed this pattern most clearly. Chou [27] also found improving self-rated health among the Chinese sample in then NHIS, suggesting that there may be some consistency in this pattern across Asian ethnicities. Finally, two studies did not any significant relationship between duration and self-rated health [39,40]. 

Part of the inconsistent findings may be due to the variety of models examined with self-rated health as an outcome. Studies included variables for cohorts [33], social connection [37], occupational class [40] or social status [35]. While the variety of models limits direct comparison of self-rated health and duration trends, they also highlight the sensitivity of the self-rated health and duration patterns to different model specifications. 

## 4. Discussion

### 4.1. Summarizing the Empirical Support for the Negative Acculturation Theory

Duration, or years in the United States, is often used as a proxy for the negative acculturation effect, which predicts worsening health with longer residence in the United States. This negative duration pattern was seen most consistently among Asian immigrants in body weight. In disability and chronic disease, there were notable differences across measures and groups. There was little support for the theory in self-rated health. There are some caveats that highlight the sensitivity of the duration patterns and limit the full support of the negative acculturation theory, however. 

First and most significantly, the findings for health behaviors do not wholly support the pathways proposed by the negative acculturation theory. There was little indication that behaviors worsen with longer duration. Longer-term Asian immigrants exercise and go to the doctor more and have both better and worse diets. The health behaviors that appear to be related to increased duration oppose one another, leaving little clarity about the nature of the actual health outcomes that can be predicated on these behaviors. Studies that did examine health behaviors as a mediator did not find that the duration effect was fully explained after including smoking or physical activity [4,32,33]. 

Secondly, there is variation in duration patterns across age, Asian ethnicity, and gender. Age was a significant moderator of the relationship between duration and disability outcomes in the two studies that examined its effect. Age is hypothesized to reduce the duration effect among older immigrants, as the aging process can introduce health risks that supersede any duration-related decline [42]. We find only mixed support for this hypothesis, as the exact nature of the age effect modification varied by measure. Worsening health with duration was seen among younger immigrants in some outcomes (*i.e.*, mobility disability), but among older mmigrants in others (*i.e.*, work disability). Nonetheless, differences across age groups suggest that duration patterns have a differential impact across the lifecourse.

There were also differences across Asian ethnicity. While both Korean and Chinese samples had higher body weights with longer duration, Koreans immigrants seemed to display an inverted-U shape between duration and BMI, while Chinese immigrants displayed a monotonic increase in obesity [29,31]. Differences in the analytic models and statistical tests limit the direct comparability of these results, however, and more research is needed to compare duration patterns across different Asian ethnicities. Some Asian ethnicities did not show any relationship between duration and health outcomes while others did. For example, the odds for obesity rose with duration for immigrants from Central Asia, but not those from Southeast or South Asia [6]. Some health outcomes were only tested among certain Asian ethnicities, so we do not know whether the findings are applicable to other groups. For example, self-reported chronic conditions were examined among Filipinos, Chinese, and Japanese only. 

There were also gender differences in duration patterns. For example, one study found that women had higher risk for obesity with duration while men had higher risk for overweight [28]. Another study found physical inactivity to increase only among women [20]. 

Another caveat was the variation within physical health conditions across different measures. In disability, mobility disability actually showed an inverse duration pattern compared to physical disability in the 2000 Census. A general count of chronic conditions seemed to support the negative acculturation theory, but the same patterns were not upheld when examining specific chronic conditions. The inconsistency within a single health outcome raises doubt about the robustness of the duration patterns for any one measure.

Another caveat is that some studies indicated a non-linear relationship between duration and health. This is in contrast to the expected negative linear pattern implied by acculturation theory. For many studies, it seemed that duration effect had a threshold effect rather than a linear progression [3,35,37]. Other studies demonstrated a curved or inverted-U duration pattern [9,29]. These alternative patterns imply more complex integration patterns than a natural and inevitable progression towards an Americanized ideal. While many studies did find a monotonic gradient across the duration dummies such that the coefficients increased relative to the reference group, there were few tests of trend (one exception was Kandula and Lauderdale [20]). What is more, duration groups were often compared to a U.S.-born reference group and not to one another, so it is unknown whether the duration groups significantly differed from one another. As a result, the linear patterns across duration categories for the majority of studies are inconclusive. 

A final caveat is that large sample samples may have created statistically significant differences among small effect sizes. This is especially true for the studies using the Census. The Cho and Hummer sample size was over 200,000 [19] and Ro and Gee sample size was just under 300,000 [36]. Repeated cross-sectional surveys, such as the NHIS, were combined across several waves to increase sample size. This limitation is tempered somewhat by other findings that seem to support worsening health among smaller samples, such as the FACES study or the CHIS. Nonetheless, the potential over-powering by large sample sizes is a valid concern and we thus suggest some caution when interpreting some of the findings

### 4.2. Alternatives to the Negative Acculturation Theory

The empirical heterogeneity in the duration and health relationship implies that Asian immigrant health does not uniformly decline with longer residence in the United States. The negative acculturation theory, in its most widely understood form, is not solely sufficient to explain duration patterns for all health outcomes and Asian immigrant populations. This section proposes alternative explanations that better encompass contextual factors for three empirical patterns between duration and health uncovered in the review: (1) heterogeneity across age, gender and sex; (2) health decline without worsening health behaviors; (3) inconsistency across health outcomes and measures. 

#### 4.2.1. Heterogeneity Across Age, Gender and Sex

If we accept that the duration is a proxy for acculturation processes that are driven by individual behavioral changes, it seems that not all groups “acculturate” to the same degree and at the same rate. Factors such as age, Asian ethnicity, and gender seem to moderate the Americanization process and coinciding adoption of Westernized behaviors. These characteristics can produce unique factors that protect against the potential health risks that accompany acculturation. For example, social support is a protective factor in health status among Asian immigrants [43]. Immigrant women benefit more from social support than men, however, potentially underlying group differences across gender [44]. 

There are theoretical frameworks for differential acculturation in the sociological literature. In their theory of segmented assimilation, Portes and Zhou [45] suggest that social stratification propels unique acculturation trajectories across groups. Certain immigrant groups are categorized into different strata of the American social system and will thus “acculturate towards” different reference groups. The theory questions the White, Anglo-Saxon ideal and emphasizes that there is more than one way of “becoming American” [46]. While segmented assimilation has primarily focused on differences in acculturation by racial groups, it can be extended to other salient group differences among immigrants, such as mode of entry or socioeconomic background. This ultimately means that US-born comparison groups should be selected with theoretical justification. Empirical tests of segmented assimilation have been limited to educational outcomes among children of immigrants but have only found limited support for the idea [46]. Future research could expand empirical tests of the theory to additional datasets and populations and consider health outcomes explicitly.

Other sources of differential acculturation may arise from country of origin characteristics. Countries of origin differ in their social patterning of disease and wellness, which bears on the decision to migrate, determines health at migration, and interacts with the social determinants of health of the receiving countries [47]. One example of this is the differing levels of the epidemiologic and nutrition transitions across country of origin. The epidemiologic transition is the shift from higher mortality from infectious diseases to a higher prevalence of chronic and degenerative conditions. The nutrition transition is complementary and is the shift from undernutrition to diets high in fats and processed foods [48]. Some developing Asian countries, such as China, India, and Vietnam, are in the midst of such transitions [49] and immigrants from these countries may have a higher likelihood for poorer health with duration as they encounter new behavioral and environmental risks after migration. In contrast, other more developed Asian countries, such as Japan and Korea, have already completed these transitions [49]. Immigrants from these countries may have already been exposed to similar environments in their home countries and may not experience health deterioration with longer U.S. exposure. 

Another source of group difference among immigrants is geographic location after migration. Where immigrants ultimately settle determines the social networks, ethnic resources, economic opportunities, and the local food environment available to them. Living with co-ethnics appears to be health-protective for Asian immigrants [25,50], suggesting that neighborhood characteristics may moderate duration patterns as well. Neighborhoods populated with other Asian immigrants may reduce exposure to the United States and related health risks and we would thus expect a weaker relationship between duration and health in these groups. Duration patterns may be unique for immigrants settling in neighborhoods with fewer co-ethnics or in new destination areas without a long history of migration.

#### 4.2.2. Health Decline without Worsening Health Behaviors

Although health behaviors did not worsen with duration, physical health still declined in several studies, especially for body weight. One possibility is that the health behaviors were poorly measured; the validity of self-reported health behaviors has been long-questioned [51,52,53]. All of the studies included in this review relied on self-reported information, which leaves open the possibility that behaviors may have indeed been affected with duration, but this pattern was not detected because of measurement error. Future research should clarify these patterns with stronger health behavior data.

Apart from measurement error, the inconclusive findings for health behaviors may also suggest that Asian immigrants face additional health risks that accumulate with longer U.S. residence that are distinct from individual-level behavior change. One possibility is stress; migration is an inherently stressful experience; immigrants contend with language barriers, blocked labor market opportunities, guilt over leaving family members in origin countries and concerns over immigration status. These stressors have been associated with worse health, independently of other migration characteristics, such as English proficiency [54]. With longer duration, immigrants also have more exposure to discrimination on the basis on their race or nativity. Racial discrimination has been consistently associated with poorer health outcomes among Asians, such as higher BMI, oorer self-rated health, and chronic conditions [55]. Connecting this to duration, stressors can exert a cumulative effect, such that those with the longest duration should have the poorest health. Indeed, longer-term Asian immigrants reported more stress than shorter-term immigrants [15]. While a majority of the studies considered duration as a proxy for acculturation, there were a few that conceptualized duration in light of cumulative stress [16,37].

The impact of migration-related stressors can ebb and flow with U.S. residence, however, suggesting that the relationship between duration and health outcomes need not be linear. Hurh & Kim [56] suggest there are two critical stages of vulnerability in regard to immigrant stress. The earliest stage immediately follows migration and is the result of stressors from migrating, such as language barriers, underemployment and social isolation. The latter stage occurs 11–15 years after migration and is the result of social marginality as immigrants confront structural and racial barriers that prohibit their full integration into the native-born White mainstream. They found some support for theory among male Korean immigrants; the prevalence of poor mental health was highest in the earliest years of residence and again at the later years. This approach has not been widely applied in national-level studies and physical health outcomes, but can serve as a useful example for future stress and coping models.

Another possibility for health decline over duration in the absence of worsening health behaviors may be due to cohort differences. Duration and cohort effects are confounded in cross-sectional data; differences between duration groups may not actually be due to increased U.S. residence, but because the groups are unique cohorts that possess distinct features [57]. It is possible that the longer duration groups may have poorer demographic profiles or health status to begin with. Thus, their worse health patterns relative to the more recent duration groups are reflecting their compositional disadvantage and not any U.S. residence-related health decline. 

#### 4.2.3. Inconsistent Patterns across Health Outcomes and Measures

There were inconsistent patterns across the four health outcomes and even more inconsistency when comparing different measures within the same health outcome. This suggests that duration can encompass multiple pathways to health that have a variety of health outcomes. Negative acculturation assumes that Americanization is bad for health, yet there are reasons to believe that longer duration can foster beneficial adaptation processes that can actually bolster immigrants’ health status. These beneficial pathways can cancel out health-harming ones, thus driving the inconsistent findings observed in this review.

Chief among these benefits is English language ability. Several studies have shown that immigrants improve in their English ability with longer residence in the United States [58,59]. Better English proficiency has been associated with better self-rated health and quality of life [59,60]. Interestingly, English preference and proficiency do not have the same effect on health. English preference, which may indicate cultural preference or “acculturation”, was not associated with any improvement in health while proficiency was significantly associated with better health [59]. The difference between English proficiency and preference suggests that improving English reflects one’s functional ability to navigate new environments. This may be especially true in regard to health care, as non-English speaking individuals are less likely to receive medical services and have poorer medical comprehension [61,62].

Another beneficial adaptation process is related to income; economists have long asserted that immigrants have higher earnings with longer duration in the United States, even surpassing native-born US-Whites [63]. The pattern is attributed to immigrants’ improving labor market characteristics, such as language ability, familiarity with the U.S. labor markets, and professional ties. Increasing wages would suggest that immigrants are accruing health-promoting resources with longer duration. Immigrants are not only earning higher incomes, but may be reaping the health benefits of the mediating factors that underlie the wage patterns (ie, better English ability, more social networks, better jobs). The possibility that duration may lead to better health was borne out in some of the empirical work [22,27,33]. Other studies examining Latino immigrants have found a positive effect of duration as well [64]. 

### 4.3. Future Research

#### 4.3.1. Pathways and Specific Health Outcomes

The notion that duration can encompass multiple processes highlights the need for future work that examines pathways between years in the United States and specific health outcomes. The current literature has few tests of mediation and their results are inconclusive. For example, neither occupational status nor health behaviors impacted the duration patterns in BMI [4,33]. There are no significant duration effects in diet after neighborhood characteristics are accounted for [26]. Mediation analyses that are guided by theoretical frameworks can offer important insight into exactly what processes are unfolding with longer residence and what the health implications are. 

Likewise, future duration research should be clear about the pathways leading to specific health outcomes. The empirical review revealed heterogeneity across different health measures, suggesting duration-related processes may be more salient to some outcomes than others. Comparison across health outcomes may be very helpful in this regard, as we can leverage contrasting results to understand broader phenomena. One study compared obesity and self-health health and found dissimilar duration patterns, such that Asian immigrants displayed increasing overweight/obesity with longer residence while there was a slight improvement self-rated health [33]. The authors suggested the divergence could be attributed to a lag between the health effects of obesity or from changing norms, such as immigrants who experience improvement in food availability feel favorably towards weight gain in the United States. Future studies could expand on such comparisons to better define unique pathways to unique health outcomes. 

#### 4.3.2. Confounding with Time-Based Measures

As previously discussed, one of the biggest methodological limitations with the duration effect is its potential confounding in cross-sectional data with cohort effects. While some studies have begun to acknowledge this bias and control for cohort of entry [30,33], the majority of other studies have not, which raises some doubt about the validity of duration patterns. The few studies that have explicitly examined cohorts have found significant cohort differences, although they do not appear to completely account for duration effects. At minimum, research should be mindful of such bias and account for them in analyses. Alternatively, researchers could develop theoretical connections between cohorts and duration, such as how conditions of entry impact subsequent integration experiences. 

Age of entry is another potential confounder of duration patterns, as the longest duration category is also likely to have the highest proportion of immigrants who migrated at younger ages. The age at which one enters the United States shapes subsequent social and educational experiences, which in turn have important implications on the developmental context of disease [6]. Those migrating at younger ages display higher rates of mental disorders and obesity than those who migrated at older ages [65,66]. Few studies have attempted to untangle age of entry from duration effects. One study, however, examined the two variables separately in the same multivariate model and concluded that each exhibited unique patterns with self-rated health [37]. 

Similar confounding problems exist with age and period effects. The most ideal way to separate out confounding among such time-based measures is to utilize longitudinal data. For example, Newbold examined Canadian longitudinal data to disentangle duration effects from cohort effects and found both factors to have a significant impact on self-rated health. All immigrants in Canada displayed worsening self-rated health over a seven year period, but cohorts entering between 1990–1994 showed the steepest decline [67]. There have not been comparable studies in the U.S., although some studies have used U.S. longitudinal data to examine immigrant health changes over time [32,68]. Harris *et al.* [68] followed a cohort of adolescents through adulthood and found immigrants to have a slower rise in BMI over increasing age than their U.S.-born counterparts. While this finding does not preclude a duration effect per se, it illustrates the need for future research to determine whether immigrants display unique health trajectories. 

These methodological issues underscore a deeper problem with the duration-related literature: in order to accurately assess immigrant health trajectories, researchers need a relevant comparison group from the country of origin to determine causation. Current research compares the health status of the foreign-born to the U.S.-born to determine duration patterns, yet this comparison does not accurately capture the effects of U.S. exposure. The best analysis would be a longitudinal comparison between migrants and their non-migrating counterparts in their country of origin. The availability of such data is extremely limited, yet there are some promising datasets that might be used in this regard, such as the Health and Retirement Study (HRS) and its international counterparts in Mexico, South Korea or China. 

#### 4.3.3. Alternative Duration Trends

While theoretical hypotheses of duration imply some kind of temporal pattern, duration is often devoid of functional form in multivariate analysis. Most often, duration categories are entered as separate indicator variables with the U.S.-born as baseline and their individual coefficients are compared to one another. Future research should incorporate a range of alternative coding patterns for years in the United States to consider a wider variety of duration patterns. For example, if researchers are hypothesizing a threshold in which risks from duration are especially high, duration should be dichotomized or entered as a quadratic function. Alternatively, if researchers conceptualize duration as a proxy for a cumulative risk factor, it should be entered linearly, or as ordinal variables with a corresponding test for trend.

## 5. Conclusions

This paper reviewed 28 studies to examine empirical support for the negative acculturation theory. Overall, it does not appear that the negative acculturation effect, in its most widely understood form, is sufficient to explain Asian immigrants’ health patterns with longer residence in the United States. The overall findings of the review echo many of the criticisms that have been generally leveled at acculturation theory: there is group heterogeneity in assimilation patterns; there are multiple dimensions of assimilation, some of which are beneficial for health and others that are not; assimilation is not linear but influenced by context. In some respects, this review joins the growing body of literature that has problematized classic acculturation theory as the primary interpretation of immigrant health patterns. Yet this review has also demonstrated that immigrants’ health is indeed affected by residence in the United States and that there is still much to be learned about what is driving these trends. The assumption that duration patterns approximate negative acculturation has hampered significant investigations into the role of length of U.S. residence on immigrant health. In the future, researchers could consider alternative pathways for duration that incorporate contextual factors and produce multiple health outcomes.
